# Evaluating the sustainability of a cancer screening intervention through a PRISM: The PreView experience

**DOI:** 10.1016/j.pmedr.2021.101443

**Published:** 2021-06-10

**Authors:** Judith Walsh, Michael Potter, Elizabeth Ozer, Ginny Gildengorin, Natasha Dass, Lawrence Green

**Affiliations:** a Division of General Internal Medicine, Department of Medicine, University of California San Francisco, San Francisco, CA 94143, USA; b Department of Family and Community Medicine, University of California San Francisco, San Francisco, CA 94143, USA; c Division of Adolescent and Young Adult Medicine, Department of Pediatrics, University of California San Francisco, San Francisco, CA 94118, USA; d Office of Diversity and Outreach, University of California San Francisco, San Francisco, CA 94143, USA; e Dell Medical School, The University of Texas at Austin, Austin, TX 78712, USA; f Helen Diller Comprehensive Cancer Center and Department of Epidemiology and Biostatistics, University of California San Francisco, San Francisco, CA 94143, USA

**Keywords:** Prevention, Cancer screening, Maintenance

## Abstract

Although many trials of cancer screening interventions evaluate efficacy and effectiveness, less research focuses on how to sustain interventions in non-research settings, which limit the potential reach of these interventions. Identifying the factors that influence the potential for sustainability is critical. We evaluate the factors influencing sustainability of PreView, a Cancer Screening Intervention, within the context of the Practical, Robust Implementation and Sustainability Model (PRISM).

PRISM includes organizational and patient perspectives of the intervention as well as characteristics of the organizational and patient recipients. It considers how the program or intervention design, external environment, implementation, and sustainability infrastructure and the recipients influence program adoption, implementation, and maintenance. We evaluate the attempts at sustainability of PreView within the constructs of PRISM.

Encouraging patients to use PreView was more difficult outside of a clinical trial. Organizational perspectives on how the intervention fit in with other goals, patient perspectives on how the intervention is individualized (i.e. being able to choose which cancer screening to address) and focused on barriers, patient characteristics (i.e. having multiple comorbidities making cancer screening less of a priority), organizational characteristics (i.e. middle managers having competing responsibilities), external environment influences (i.e. reimbursement for achieving certain cancer screening goals), and sustainability infrastructure all affect the likelihood of PreView being sustained in clinical practice.

Despite advance planning for sustainability, adapting interventions to achieve sustainability is difficult. Lessons learned from evaluating PreView within the PRISM model can inform future sustainability efforts.

## Highlights

• Sustaining successful interventions in non-research settings is challenging.
• Practical Robust Implementation Sustainability Model (PRISM) informs sustainability.
• We evaluate factors influencing sustainability of PreView, through the lens of PRISM.
• Lessons learned from PreView and PRISM can inform future sustainability efforts.

## 1 Introduction

The United States Preventive Services Task Force (USPSTF) recommends mammography screening for women aged 50–74, cervical cancer screening for women aged 50–64, and colorectal cancer (CRC) screening for those aged 50–75 (Siu, 2016, Curry et al., 2018, Grossman et al., 2018). The American Cancer Society (ACS), American College of Physicians (ACP), and others recommend a shared decision-making approach to prostate cancer screening (PSA) (Qaseem et al., 2013, Wolf et al., 2010). Although the USPSTF previously recommended against PSA screening, current guidelines recommend a shared decision-making approach to PSA screening for men aged 55–69 (Moyer, 2012). However, few interventions address all recommended cancer screenings for which an individual is due.

With the input of patients and providers, we developed PreView, an Interactive Video Doctor intervention, that simulates interaction with a real clinician and addresses all cancer screening and discussions for which an individual aged 50–70 may be due (Arora et al., 2013). Before a scheduled visit, using an electronic tablet, patients answer questions about their screening history and readiness to undergo and/or maintain screening activity based on the Transtheoretical Model of Behavior Change (Prochaska and Velicer, 1997). Patients are asked about individual screening barriers and receive individually tailored messages via video. The clinician also receives a paper-based “Provider Alert,” including an assessment of patients’ screening status, readiness to screen, individual barriers, as well as possible responses that could help the patient overcome identified barriers to screening.

We conducted a randomized clinical trial at 6 primary care sites including 508 patients, comparing the impact of PreView with a control intervention. Primary outcomes were receipt of breast, cervical, and colorectal cancer screening as well as discussions about prostate cancer screening. Clinician-patient discussion about all cancer screenings significantly increased with PreView relative to the comparison intervention (Walsh et al., 2020).

Sustainability is the continued delivery or institutionalization of a clinical intervention or program (Moore et al., 2017). Few studies have evaluated whether an intervention, such as PreView, shown to be efficacious in a trial can be effectively sustained in non-research settings (Walsh et al., 2012). An important challenge is to balance *fidelity* to exactly what was shown to be efficacious in the context of a controlled trial with *adaptation*, revision of the intervention to better fit the circumstances and patients where it will be applied.

**Fig. 1:**
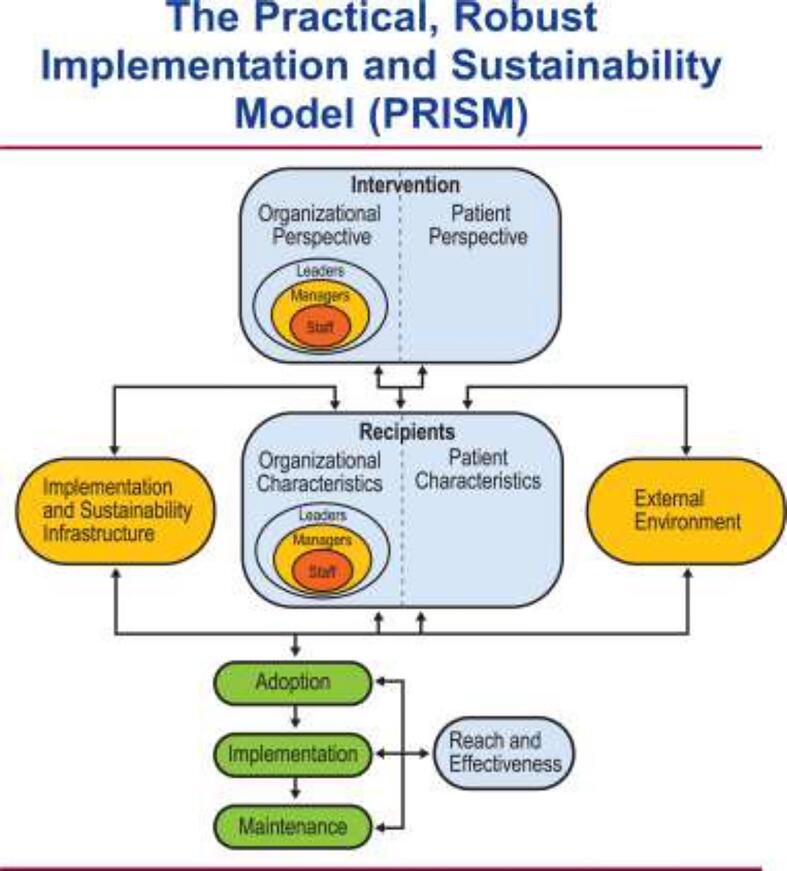
The Practical, Robust Implementation and Sustainability Model (PRISM), which illustrates how the intervention design (from an organizational and patient perspective), implementation and sustainability infrastructure, external environment, and recipients (organizational and patient characteristics) collectively impact the program's adoption, implementation, and maintenance (Feldstein and Glasgow, 2008).

Many factors can influence the potential for sustainability. The Practical, Robust Implementation and Sustainability Model (PRISM) is a comprehensive model for translating research into practice that permits evaluation of how the health program or intervention interacts with recipients to influence program adoption, implementation, maintenance, reach, and effectiveness (Fig. 1) (Feldstein and Glasgow, 2008). PRISM identifies factors to consider when translating research into real-world practice and provides guidance about measuring success and challenges. The PRISM model can provide a lens through which to evaluate the factors influencing sustainability. These include organizational and patient perspectives, characteristics of the organization and of patients, external influencing factors, infrastructure related to implementation and sustainability, as well as reach and effectiveness.

Given the importance of sustaining an intervention in clinical practice, we planned for a twelve month maintenance phase that would be conducted after completion of the PreView trial, in which we addressed the following questions: (1) How can we maintain a cancer screening intervention once the randomized controlled trial (RCT) is complete? (2) Which factors influenced the potential for sustainability of PreView? (3) What are lessons learned when trying to maintain and disseminate the results of a clinical trial?

## 2 Methods

### 2.1 PRISM description and domains

PRISM was developed with the goal of creating a comprehensive model for translating research into practice (Feldstein and Glasgow, 2008). The model considers how the program or intervention design, implementation and sustainability infrastructure, and external environment affect the program adoption, implementation, and maintenance, which are major components of reach and effectiveness (Fig. 1) (Feldstein and Glasgow, 2008). The intervention must be considered from both, the perspective of the organization and the patient. Additionally, intervention recipients include patients as well as the organization and its members. Organizational and patient recipients are influenced by the external environment and the implementation and sustainability infrastructure. All of these factors influence adoption, implementation, and maintenance which contribute to overall reach and effectiveness. Our goal was to evaluate the successes and challenges of the maintenance phase through the lens of the PRISM model.

### 2.2 Description of the PreView RCT

The results of the PreView RCT have been published elsewhere (Walsh et al., 2020). We conducted the RCT at 6 clinical sites, all of which are part of the San Francisco Bay Area Collaborative Research Network, a UCSF supported practice-based research network. The sites included 3 federally qualified health centers (FQHCs), 2 large staff model private practices, and 1 small physician owned and operated clinic associated with a larger regional health organization. Participating clinicians were told that patients would come in early to complete PreView on the tablet before their appointment and that a paper Provider Alert would be generated which the clinician could use at the visit if desired. 508 eligible patients aged 50–70 who were scheduled for an appointment were asked to come early and complete the program before their appointments. Patients were met by a research assistant who ensured that the Provider Alerts were delivered to the provider and helped patients complete post-visit questions on the tablet after their appointments. Primary outcomes of the RCT were receipt of recommended cancer screening tests or discussions about testing within recommended time intervals.

### 2.3 Maintenance phase modifications

When agreeing to participate in the study, we asked g sites to commit to participating in an RCT followed by a 12-month maintenance phase. Clinics were enrolled in a rolling fashion and all maintenance phase activity was completed by December 2018. We made initial modifications so that PreView could be freestanding: (1) We added an introductory graphic screen with pictures to show how to initiate the program, (2) eliminated many of the initial demographics, and (3) removed research references and login numbers. In consultation with leaders and staff at each location, we set up PreView in a prominent location within the clinic waiting area, and included signage inviting patients to use it. We developed methods to secure the tablet and plans for ensuring that it would remain charged. Because a printout of the patient’s response would be generated, the printer was placed behind the front desk in a secure location. During this initial phase, we saw low usage (once or twice a week or less) at all sites. To address this, we requested feedback from key stakeholders (clinic staff, providers, and patients).

#### 2.3.1 Provider and staff interviews

We conducted interviews with at least 1 provider (typically the medical director) and 2 administrative and/or staff members at each site. All interviews were conducted by 2 researchers. Interviewees were asked about their personal and their patients’ experiences with PreView, what they liked/disliked, as well as any challenges they experienced with maintaining PreView. Issues discussed included how to encourage patients to use PreView (i.e. signage), where the PreView tablet should be placed, how to best integrate PreView into clinical routine, and what level of staff involvement would be required.

#### 2.3.2 Patient focus groups

We conducted focus groups at all 6 sites. At the 3 sites where a significant proportion of the patients spoke Spanish, we conducted separate focus groups in English and Spanish. Each focus group was facilitated by 2 study investigators. Focus group participants were given information about and a demonstration of PreView, and were asked to explain what they liked and disliked about PreView. Participants provided reasons for why they think patients were not using PreView and suggestions for encouraging them to use it. They were t asked which types of signage would encourage patients to use PreView, where in the clinic patients would most likely do it (i.e. waiting area vs. exam room), and whether they would recommend it to family members.

We administered 6 focus groups in English and 3 focus groups in Spanish, in which 27 and 11 patients participated, respectively. Further, we conducted 15 key informant interviews across all 6 sites. At each site, we held 1 interview with a medical director and 1 with a clinic administrator. We conducted a total of 5 additional interviews with providers who had experience in using PreView with their patients.

Focus group and interview notes were analyzed by at least 2 investigators. Individual and recurrent themes were itemized, categorized, and evaluated within the PRISM domains.

**Fig. 2:**
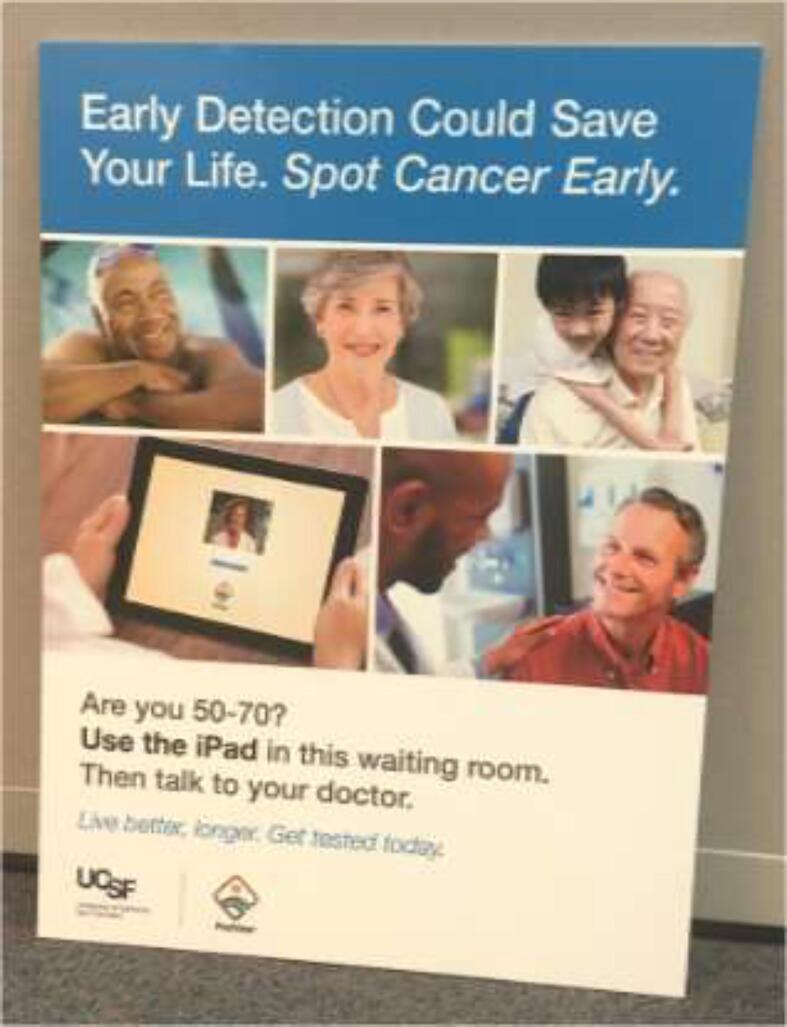
Example of a PreView poster (in English) that was developed based on focus group feedback and placed in clinic waiting rooms. We also developed posters in Spanish language.

Based on focus group input, we worked with the UCSF Marketing Department to develop appropriate messaging for posters and signage (Fig. 2). At each site, we identified the best location to host PreView, either in the waiting room or exam room. If it was placed in the waiting room, we determined the optimal location for the tablet and printer (where Provider Alerts are printed). If given to patients in exam rooms, we addressed who would be responsible for bringing the tablet to patients and how the printouts would be received. We addressed whether the tablet should be placed in an area where it would not move (i.e. locked to a desk) or on a mobile cart.

Using the Plan-Do-Study-Act (PDSA) cycle for rapid improvement (Taylor et al., 2014, Leis and Shojania, 2017), we obtained detailed input and attempted multiple adapted strategies recommended by clinical teams at each site.

PDSA, or Plan-Do-Study-Act, is an iterative, four-stage problem-solving model used for improving a process or carrying out change. It is commonly used in quality improvement initiatives.

All study procedures were approved by the UCSF Institutional Review Board.

## 3 Results

The main outcomes of the maintenance phase focused on encouraging PreView usage, rather than on receipt of cancer screening or cancer screening discussion. The number of patients using PreView remained low (sometimes once a week or less) in most clinical sites. At one small site, use was consistently higher (i.e. daily) than at all the other sites.

### 3.1 Sustainability of PreView as evaluated within PRISM domains

In our discussions with patients, providers, and staff, several barriers to PreView usage were reported. We describe these barriers within the context of PRISM domains and when feasible, strategize ways to overcome them.

#### 3.1.1 Intervention – Organizational perspective

An important question for organizational readiness was whether or not PreView was a necessary addition to the practice. Providers and staff viewed PreView as a beneficial, but perhaps not necessary addition. Another question was whether there was agreement regarding the strength of evidence upon which PreView was based. Although there was uniform agreement on the strength of evidence – with the exception of prostate cancer screening, which remained controversial throughout the study – there was concern about program-related burdens placed on frontline staff. For example, to ensure patient confidentiality, the Provider Alert needed to be printed in a secure area, which required staff to retrieve the printout to then give to either the patient or provider.

Another approach used PreView in the exam room. A perceived benefit of using PreView in the exam room is that patients have privacy and can complete the program while waiting for their doctor. However, this requires a staff member to bring the tablet to the patient, which is a challenge given their numerous other responsibilities. Finding a consistent staff member to be the “champion” for PreView was often difficult. When a “champion” was identified, the initial setup and usage of PreView was more successful.

Another factor influencing organizational interest is whether or not the organization see the results of the intervention. Since the results occurred with the doctor and the patient in the exam room, many members of the organization could not directly observe the results.

During the RCT phase, clinic staff was “used to” having the research assistant guide the patient through the intervention. Staff members expressed some concern that during the maintenance phase they had to do things that they had not previously been required to do.

#### 3.1.2 Intervention – Patient perspective

Once patients learned about PreView, they uniformly felt that it was important and that patients should know about it. Patients liked that the patient-centered program offered individually-tailored information, and felt that it was important for everyone to complete and get screening. The majority recommended PreView to their family members, and thought that it would be most effective for patients who previously declined screening. They frequently asked whether it could be done at home before their appointment.

#### 3.1.3 Recipients – Organizational characteristics

Organizational factors are considered at three levels: top management, middle management, and frontline teams. Although we saw enthusiasm for PreView at the top management level, clinical administrators at the middle management level and frontline teams often expressed less enthusiasm for integrating PreView into their already busy schedules.

Incentives can influence staff behavior. Based on suggestions from leadership and staff interviews, we carefully considered the provision of staff incentives. We were concerned that while it may increase PreView use in the short-term, the increased usage may not be sustainable. There was also organizational concern about providing incentives to staff members for promoting PreView such that it may divert them from other responsibilities. We attempted a staff incentive at 3 sites where we provided a bagel breakfast when a certain number of patients used PreView. As predicted, this transiently increased use but did not result in sustained increased usage.

An additional organizational factor relates to the physical design and level of “busyness” of the clinic setting. When PreView was set up in the waiting room, we placed it where it was somewhat obvious and inviting, but away from main traffic flow. Although we tried several types of signage, it was difficult to motivate patients to walk over to PreView. At all sites, there were other things to do in the waiting room (i.e. watch television). Patients also had access to their personal devices (i.e. cell phones), making it less likely that they would use another device available in the clinic. When a patient used PreView, we provided him/her with earphones to maintain privacy. Although we had perceived this maintenance of privacy as a benefit, there was an unexpected negative consequence: some patients were concerned that the headphones would interfere with their ability to hear if they were called for their appointment.

In another clinic, we positioned PreView at a desk in the corner of the waiting room. After noticing that patients were not walking over to the desk, we learned that waiting room space in that clinic was considered “high value real estate.” Patients were concerned that if they left their chairs to use PreView, they might lose their seat when they were done with the program. Since clinic waiting time in this setting was typically quite long, not having a place to sit was potentially a big problem, and many were not willing to risk giving up their seat. Notably, in the single location where ongoing PreView usage was most consistent, the waiting room is small, with few distractions and no public Wi-Fi.

Although the overall goal was sustainability of PreView, this was more difficult to ensure at the middle management and clinical administration level. Clinic administrators, although supportive, had other competing priorities such that the sustainability of PreView was often not at the top of the list. Similarly, frontline staff often needed to address immediate patient needs (i.e. insurance eligibility, patient check-in, timely rooming, and medication reconciliation), which meant PreView held lower priority.

#### 3.1.4 Recipients – Patient characteristics

PreView only targets individuals aged 50–70. Across practice settings, patients and providers felt that it was important that patients received appropriate screening; however, in settings that generally serve patients of higher socioeconomic status and screening rates were higher, providers and staff felt less of a need for PreView. Conversely, in settings where screening rates were lower, PreView was potentially more important in encouraging screening; however, many had other competing diseases and health concerns, placing cancer screening lower on their priority list. For example, a provider caring for a patient with poorly controlled diabetes might prioritize improving diabetic control over discussing mammography.

#### 3.1.5 External environment

There are many external features that could impact feasibility of implementing an intervention. Limited colonoscopy availability limits the impact of any colorectal cancer (CRC) screening intervention. If clinic reimbursement is partially based on achieving cancer screening thresholds, interventions focused on increasing cancer screening rates might be higher priority. One clinic in particular was not meeting its Healthcare Effectiveness Data and Information Set (HEDIS) goal for CRC screening, and chose to focus primarily on using the CRC screening module of PreView rather than offering all modules.

#### 3.1.6 Implementation and sustainability infrastructure

As described above, when developing the maintenance version of PreView, we made the following modifications to the RCT version: (1) simplified log-in, (2) made PreView accessible without a research assistant, (3) allowed module choices, and (4) provided ongoing training and support to all key staff members.

Many expressed concern that it would take a long time to complete PreView. In response, we modified the program to allow individuals to select only the modules they would like to do. Time was not only an issue for patients, but also for staff and providers. Staff expressed concern about how much they already had to do in addition to helping patients complete PreView. Providers often want patients to be roomed quickly, so adding PreView to the list of rooming tasks was not always welcomed.

We accomplished some of the adaptations during the maintenance phase, however, other changes took longer. Many patients expressed interest in completing PreView at home before their visits. Others wanted it to be available through the patient portal. Since this concern arose frequently, we worked closely with the UCSF School of Medicine Technology Enhanced Education Team to develop a website that could be accessed at home and allowed patients to relay results to their doctor. We continue to work towards making PreView accessible through the patient portal. PreView is now available at www.preview.ucsf.edu.

## 4 Conclusions

Evidence-based interventions often fail the test of translation into clinical practice. How can we effectively implement interventions in real life? Using PRISM as an evaluation tool helps identify barriers and facilitators to real-world implementation.

A prior study applied PRISM to evaluate barriers and facilitators to implementation of a CRC screening intervention at a single site (Liles et al., 2015). Investigators found that evaluation of the barriers and facilitators as well as including perspectives of multiple stakeholders led to changes in programmatic design to optimize its impact. Evaluating the impact of an intervention like PreView, which occurred in multiple sites, is more complex. However, evaluation using the PRISM domains can lead to common themes which can be useful in planning future multi-site interventions. Below, we describe some of the lessons learned from evaluating PreView through the lens of PRISM.

### 4.1 Lessons learned

#### 4.1.1 Get feedback from all relevant stakeholders

According to PRISM, organizational and patient perspectives are important. Clinicians, staff, and patients should all provide input. Even if there is support at a high level, frontline staff is doing the actual work, so their input is critical. If the intervention is going to work, it needs to work for everyone!

#### 4.1.2 Balance fidelity with adaptation

Organizational and patient characteristics will dictate which components of the intervention must stay the same and where there is room for adaptation based on local circumstances. For instance, given that PreView includes creating a Provider Alert which could be used during the appointment, we continued to offer PreView before the clinic appointment in the waiting room. However, when some of the clinics wanted to offer PreView in the exam room, we tried that approach as well.

#### 4.1.3 Consider impact of the intervention on staff and providers

We understand that everyone is busy and the clinic has an existing workflow before implementing this intervention. Successful interventions fit in with existing clinic flow. Think carefully about creating additional work for staff and providers and about mitigating the need for additional work.

#### 4.1.4 Have a clinic champion

Previous research supports the importance of having a champion in order to successfully sustain change (Shaw et al., 2012, Holland et al., 2010). Having one key individual who believes in the intervention and is part of the organization’s ongoing work can facilitate change. It is often hard to find, but if you do, it makes things run more smoothly.

#### 4.1.5 Be flexible. If one thing does not work try another

Although PreView was developed with the goal of focusing on all cancer screenings for which a patient is potentially due, there was concern that completing all modules takes too much time. In addition, some clinical sites were focused on increasing one type of cancer screening, typically colorectal. Thus, we modified the original program such that participants could complete only one or several modules of their choosing.

#### 4.1.6 Solving one problem may create another

With the challenge of ensuring patient privacy in the waiting room, we provided headphones and covers for patient use. However, after seeing some patients’ reluctance to use headphones, we learned that they were concerned about not hearing their name called for their appointment. If something is not working as anticipated, ask why.

#### 4.1.7 If something is not working, address it quickly

Things move quickly in a busy clinic. Something that is not working for one person often will not be working for others. Addressing concerns quickly avoids repetition of problems and shows receptivity to concerns.

In conclusion, evaluating our sustainability plan and experience through PRISM provided valuable insight into the barriers and facilitators of implementing and sustaining a novel cancer screening intervention. Future implementation efforts should continue to evaluate the potential for implementation and sustainability using the PRISM domains of intervention/program design (from an organizational and patient perspective), recipients (organizational and patient characteristics), implementation and sustainability infrastructure, and external environment *before* developing the implementation plan as well as *throughout* the implementation phase to allow for ongoing program modifications. Ultimately, this development of “practice-based evidence” will inform “evidence-based practice” (Green, 2014, Green, 2008).

## Funding

Funded by the 10.13039/100000054National Cancer Institute: R01 CA158027.

## CRediT authorship contribution statement

**Judith Walsh:** Conceptualization, Visualization, Formal analysis, Funding acquisition, Investigation, Methodology, Writing - original draft, Writing - review & editing. **Michael Potter:** Conceptualization, Investigation, Methodology, Writing - review & editing. **Elizabeth Ozer:** Investigation, Methodology, Writing - review & editing. **Ginny Gildengorin:** Data curation, Formal analysis, Writing - review & editing. **Natasha Dass:** Data curation, Project administration, Formal analysis, Writing - review & editing. **Lawrence Green:** Conceptualization, Formal analysis, Writing - review & editing.

## Declaration of Competing Interest

The authors declare that they have no known competing financial interests or personal relationships that could have appeared to influence the work reported in this paper.
